# The anti-interleukin-6 antibody ALD518-P18 mitigates murine kidney ischemia–reperfusion injury

**DOI:** 10.1038/s41598-026-54952-9

**Published:** 2026-05-27

**Authors:** Anjan K. Bongoni, Mark Biondo, Jennifer L. McRae, Evelyn J. Salvaris, Nella Fisicaro, Fenella Muntz, Tony Rowe, Adriana Baz Morelli, Peter J. Cowan

**Affiliations:** 1 Immunology Research Centre, St. Vincent’s Hospital, PO Box 2900 Fitzroy 3065, Melbourne, VIC Australia; 2 CSL Ltd, Melbourne, VIC Australia; 3 Bioresources Centre, St. Vincent’s Hospital, Melbourne, VIC Australia; 4 Department of Medicine, University of Melbourne, Melbourne, VIC Australia

**Keywords:** Kidney ischemia–reperfusion injury, Interleukin-6, IL-6 trans-signalling, Anti-IL-6 antibody, Inflammation, Kidney transplantation, Diseases, Immunology, Medical research, Nephrology

## Abstract

Interleukin-6 (IL-6) is a key pleiotropic cytokine implicated in kidney injury; however, the therapeutic potential of IL-6 blockade against acute ischemia–reperfusion injury (IRI) remains uncertain, with previous murine studies yielding conflicting results. This study investigated the protective effects of the high-affinity chimeric anti-IL-6 monoclonal antibody, cALD518-P18, in a non-transplant mouse model of warm renal IRI (22 min ischemia, 24 h reperfusion). Male C57BL/6 mice received a single 40 mg/kg dose of cALD518-P18 or an isotype control, administered either intraperitoneally 30 min before ischemia or intravenously immediately after ischemia. At 24 h, control mice exhibited significant kidney dysfunction (serum creatinine, urea), injury, and inflammation, consistent with elevated pro-inflammatory IL-6 trans-signalling (soluble IL-6R (sIL-6R) and tubular STAT3 phosphorylation (pSTAT3)). Treatment with cALD518-P18, in both regimens, significantly protected the kidneys from IRI, demonstrating improved renal function and reduced tissue damage. The antibody attenuated inflammation, endothelial activation and pSTAT3 signalling, significantly downregulating gene expression of the acute tubular stress marker NGAL. These findings confirm the pathogenic role of IL-6 and highlight its early blockade with cALD518-P18 as a promising therapeutic strategy to improve outcomes in acute kidney injury and transplantation.

## Introduction

Interleukin-6 (IL-6), a pleiotropic cytokine that acts on multiple cell types including T cells, B cells, neutrophils and endothelial cells, can induce either pro- or anti-inflammatory responses depending on the context^1^. There are three known pathways of IL-6 signalling^2^. When IL-6 binds to membrane-bound IL-6 receptor (mIL-6R), the IL-6/mIL-6R complex recruits glycoprotein 130 (gp130) on the same cell (classical signalling) or on neighbouring cells (cluster signalling). In the kidney, mIL-6R is expressed only in podocytes and glomerular mesangial cells^3^, restricting classical signalling to these cell types. IL-6 can also bind soluble IL-6R (sIL-6R) cleaved from the membrane by the protease A Disintegrin And Metalloproteinase 17 (ADAM17), and the circulating IL-6/sIL-6R complex activates gp130 on cells it encounters (trans-signalling). Because gp130 is ubiquitously expressed, trans-signalling can occur in any cell type. Regardless of the IL-6 signalling mode, homodimerization of gp130 can activate a range of intracellular signalling pathways including JAK-STAT, MAPK, and PI3K/Akt^4^. Recent evidence suggests that IL-6 trans-signalling is a particularly important component of the pathogenetic mechanism in various inflammatory diseases and conditions^2^, including ischemic acute kidney injury (AKI)^3^.

Clinical trials using antibodies to IL-6 or IL-6R have produced promising results in the treatment of chronic active antibody-mediated rejection (caAMR) of kidney allografts^5^. However, there may also be a role for such antibodies in the acute setting of kidney ischemia–reperfusion (IR) injury (IRI), an inflammatory process that significantly impacts renal function in a range of clinical settings including kidney transplantation^6^. IRI has both acute and chronic detrimental effects on the kidney. IL-6 levels increase markedly during renal IR, triggering immune responses and initiating tissue repair mechanisms^7^. Neutrophils, as early responders to IL-6, help to clear cellular debris but can exacerbate injury through the release of reactive oxygen species and proteolytic enzymes^1^. Elevated IL-6 facilitates the differentiation of naïve T cells into pro-inflammatory Th17 cells^8^, enhancing the recruitment of immune cells to the site of injury. IL-6 also promotes B cell activation and antibody production^9^, amplifying the immune response. This coordinated effort is crucial for addressing acute injury, but excessive IL-6 production can lead to chronic inflammation and tissue fibrosis^10^.

Several mouse studies have investigated the impact of modulating IL-6 activity on renal IRI. IL-6 knockout mice showed less severe injury than wild-type (WT) mice^11^, and reconstitution of knockout mice with WT bone marrow restored susceptibility^12^, indicating a pathogenetic role for IL-6 in renal IRI. However, anti-IL-6 antibody treatment in murine warm renal IRI models has produced conflicting results, with reports that anti-IL-6 was protective^12^, had no effect^11^, or even slightly exacerbated renal IRI^13^. These apparent discrepancies may be partly explained by differences in the type and/or dose of the antibodies used, compounded by variations in the models employed, including mouse strain and age, mode of clamping (unilateral or bilateral), and timing of treatment and/or analysis. Adding further complexity, plasmid-mediated expression of recombinant IL-6/sIL-6R, which would be expected to promote pro-inflammatory trans-signalling, had the surprising effect of protecting mice against renal IRI^14^.

The aim of this study was to use our well-established mouse model of unilateral renal IRI, in which we have previously demonstrated protection by several anti-inflammatory molecules^15–19^, to assess the protective efficacy of anti-IL-6. We also sought evidence of IL-6 trans-signalling in this model. Clazakizumab is a humanized rabbit anti-IL-6 monoclonal antibody (mAb) currently in advanced clinical development^20^. Because clazakizumab does not cross-react with mouse IL-6, we employed a specific chimeric antibody, designated ALD518-P18-muG1[N297A] and referred to hereafter as chimeric (c)ALD518-P18. This antibody is a variant of ALD518-P18^21^, which is itself a human antibody derived from clazakizumab and engineered with complementarity-determining regions (CDR) mutations to enhance binding and neutralization of murine IL-6. The cALD518-P18 version combines the human variable domains from ALD518-P18 with mouse IgG1 constant domains (Fc, CH1, and CL). Additionally, it incorporates the N297A mutation in the Fc region to eliminate N-linked glycosylation, thereby minimizing potential effector functions such as antibody-dependent cell-mediated cytotoxicity (ADCC). Our results showed that cALD518-P18 treatment, whether administered before ischemia or after reperfusion, significantly protected mice from renal IRI, maintaining renal function and reducing tubular injury and inflammation.

## Results

### cALD518-P18 treatment before or immediately after ischemia reduces renal IRI in mice

We used a mouse model of unilateral warm renal IRI^16^ to investigate the impact of anti-IL-6 treatment on ischemic AKI. The right kidney was removed and the left renal pedicle was clamped for 22 min. Sham mice underwent the same procedure without left kidney ischemia and treatment. Kidney function (serum creatinine and urea) and tissue damage (tubular injury score) were assessed 24 h after reperfusion.

**Figure 1:**
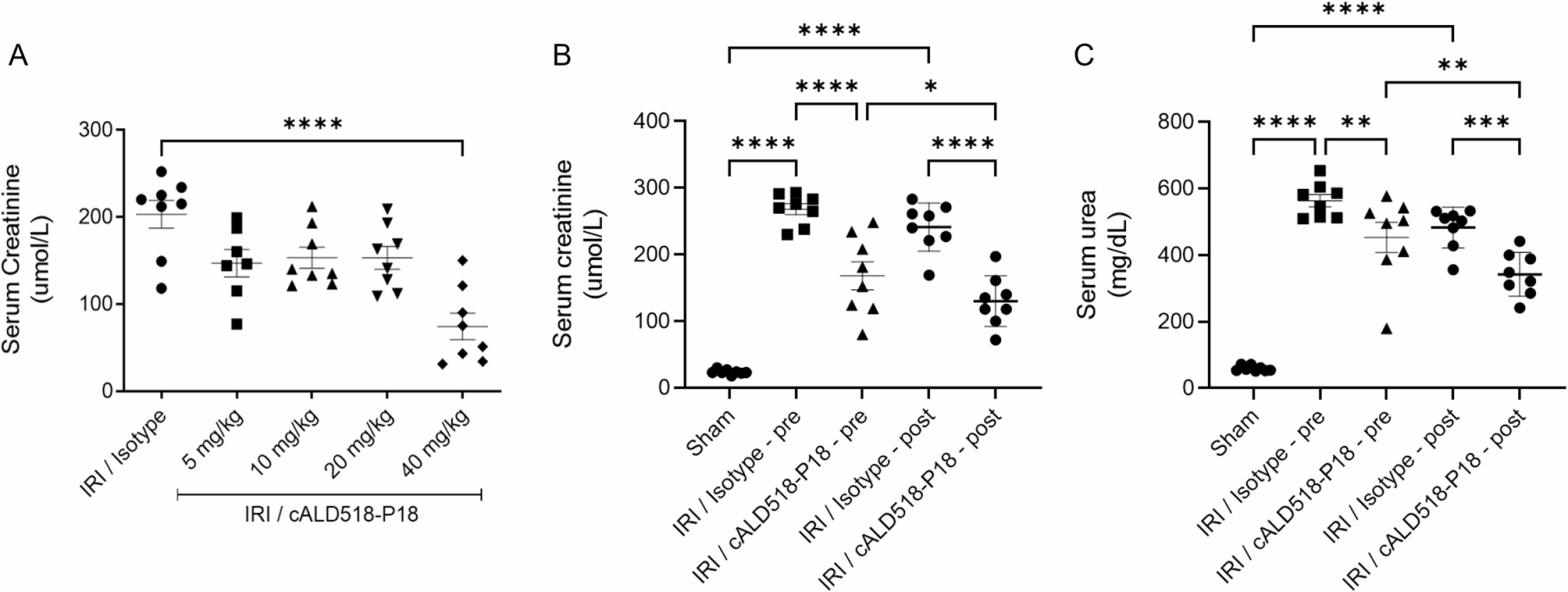
cALD518-P18 treatment preserves renal function following ischemia–reperfusion injury. (**A**) Dose–response analysis. Mice (n = 8 per group) were treated i.v. immediately post-22 min ischemia with an isotype control antibody (40 mg/kg) or cALD518-P18 at doses of 5, 10, 20, or 40 mg/kg to determine the optimal therapeutic dose. Serum creatinine levels were assessed 24 h post-reperfusion. Statistical analysis was performed using one-way ANOVA with Kruskal–Wallis and uncorrected Dunn’s multiple comparison test, **** *p* < 0.0001. (**B, C**) Evaluation of protective efficacy. Mice received 40 mg/kg of cALD518-P18 or an isotype control, administered either i.p. 30 min before ischemia or i.v. immediately after reperfusion. Renal function was determined at 24 h by measuring (**B**) serum creatinine and (**C**) serum urea. Data represent mean ± SEM (n = 8 per group). Significance was determined by one-way ANOVA followed by uncorrected Fisher’s LSD test to perform pre-dedined, planned comparisons (*p < 0.05, **p < 0.01, ***p < 0.001, ****p < 0.0001).

First, to establish an effective dose of cALD518-P18, we conducted a dose–response study. Mice were subjected to renal IRI and treated i.v. immediately post-ischemia with 10, 20, or 40 mg/kg of cALD518-P18, or 40 mg/kg of an isotype-matched control antibody. The 40 mg/kg dose provided significant protection, as measured by a reduction in serum creatinine (*p* < 0.0001 versus IRI/Isotype), while lower doses were not significantly effective (Fig. 1A). Therefore, we selected the 40 mg/kg dose for the definitive experiments presented in this study. In these experiments, mice received 40 mg/kg of cALD518-P18 (IRI/cALD518-P18) or an isotype-matched control mAb (IRI/Isotype), either i.p. 30 min before ischemia or i.v. immediately after reperfusion.

Mice in the IRI/Isotype groups (i.e. with isotype control mAb treatment pre- or post-ischemia) exhibited significant increases in serum creatinine (Fig. 1B, both *p* < 0.0001 versus Sham) and urea (Fig. 1C, both *p* < 0.0001 versus Sham), indicating markedly reduced renal function at 24 h. Treatment with cALD518-P18 either before or after ischemia provided significant protection against renal dysfunction. Mice receiving cALD518-P18 before ischemia showed notably lower creatinine (Fig. 1B, *p* < 0.0001 versus IRI/Isotype) and urea (Fig. 1C, *p*  < 0.01 versus IRI/Isotype), as did mice treated after ischemia (Fig. 1B , *p* < 0.0001 for creatinine and Fig. 1C, *p*  < 0.001 for urea, both versus IRI/Isotype). Post-ischemia i.v. treatment was more effective than pre-ischemia i.p. treatment (creatinine: *p* < 0.05 IRI/cALD518-P18-post versus IRI/cALD518-P18-pre, Fig. 1B; urea: *p* < 0.01 IRI/cALD518-P18-post versus IRI/cALD518-P18-pre, Fig. 1C).

**Figure 2:**
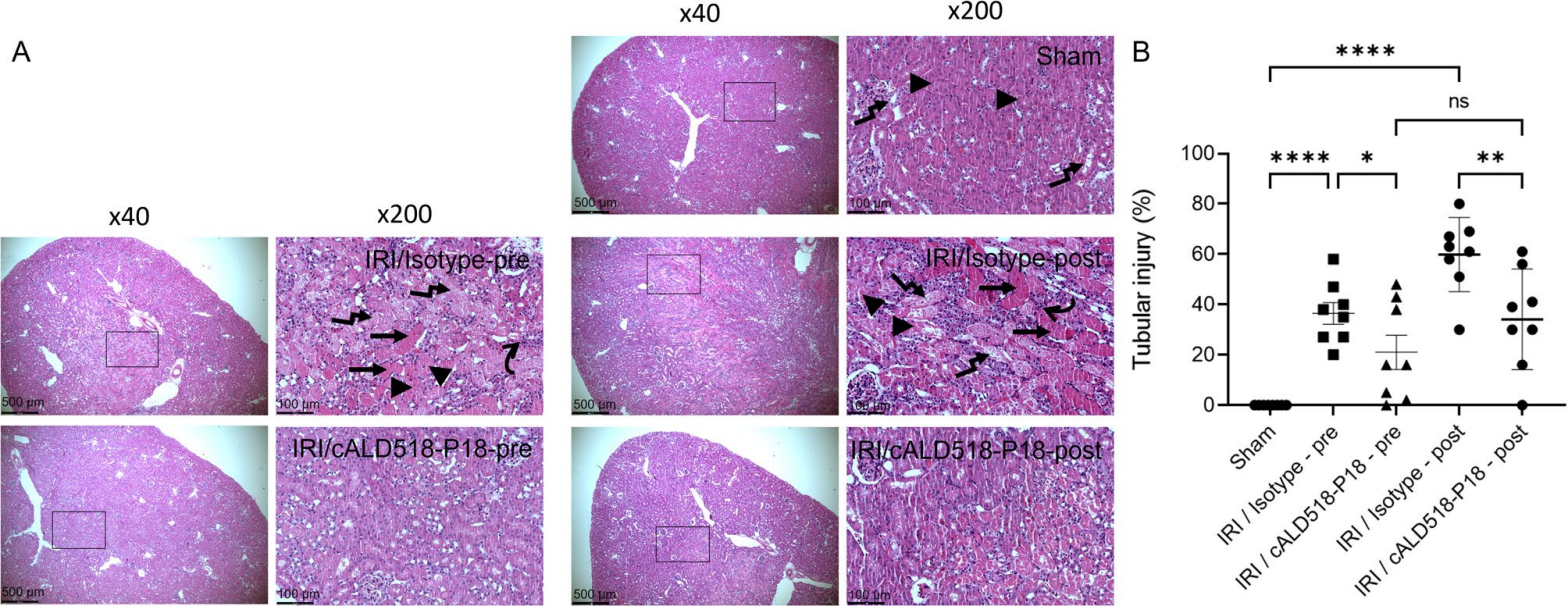
cALD518-P18 treatment reduces histological tubular injury after renal ischemia–reperfusion. Histological changes were assessed 24 h post-reperfusion by measuring tubular injury in kidney sections. (**A**) Representative images of hematoxylin and eosin (H&E) stained sections at low (× 40, scale bar: 500 μm) and high magnification (× 200, scale bar: 100 μm). Square boxes in low-magnification images indicate the areas captured at high magnification. In healthy kidneys (Sham, × 200), normal renal tubules with intact epithelial cells (indicated by arrowheads) and an intact brush border (indicated by elbow arrows) appear as a pink, somewhat fuzzy lining within the tubule lumen. Following IRI, Isotype control groups exhibit areas of necrosis and dilation (indicated by arrowheads), cast formation consisting of sloughed epithelial cells and debris (indicated by arrows), loss of brush border in the tubule cells (indicated by elbow arrows), and infiltration of inflammatory cells (indicated by curved arrows). (**B**) Quantification of tubular inury scores. IRI/cALD518-P18 mice showed significant reduction in injury scores compared to IRI/Isotype in both pre- and post-ischemia treatment groups. Data are presented as mean ± SEM (n = 8 per group). Statistical significance was determined by one-way ANOVA followed by uncorrected Fisher’s LSD test to perform pre-dedined, planned comparisons (*p < 0.05, **p < 0.01, ****p < 0.0001; ns, not significant).

Histological analysis confirmed the protective effects of cALD518-P18. IRI mice treated with isotype control mAb either before or after ischemia exhibited severe left kidney damage at 24 h, characterized by tubular dilation, necrosis, luminal congestion, and eosinophilia (Fig. 2A). This was confirmed by tubular injury scoring using a semi-quantitative method (Fig. 2B, IRI/Isotype-pre and IRI/Isotype-post both *p* < 0.0001 versus Sham). Kidneys from IRI mice treated with cALD518-P18 before or after ischemia showed a significant reduction in tubular injury (Fig. 2B, *p*  < 0.05 and *p* < 0.01 versus respective IRI/Isotype controls). The degree of tubular injury was similar in the IRI/cALD518-P18-pre and -post groups.

### cALD518-P18 attenuates innate immune cell infiltration of the kidney following renal IRI

**Figure 3:**
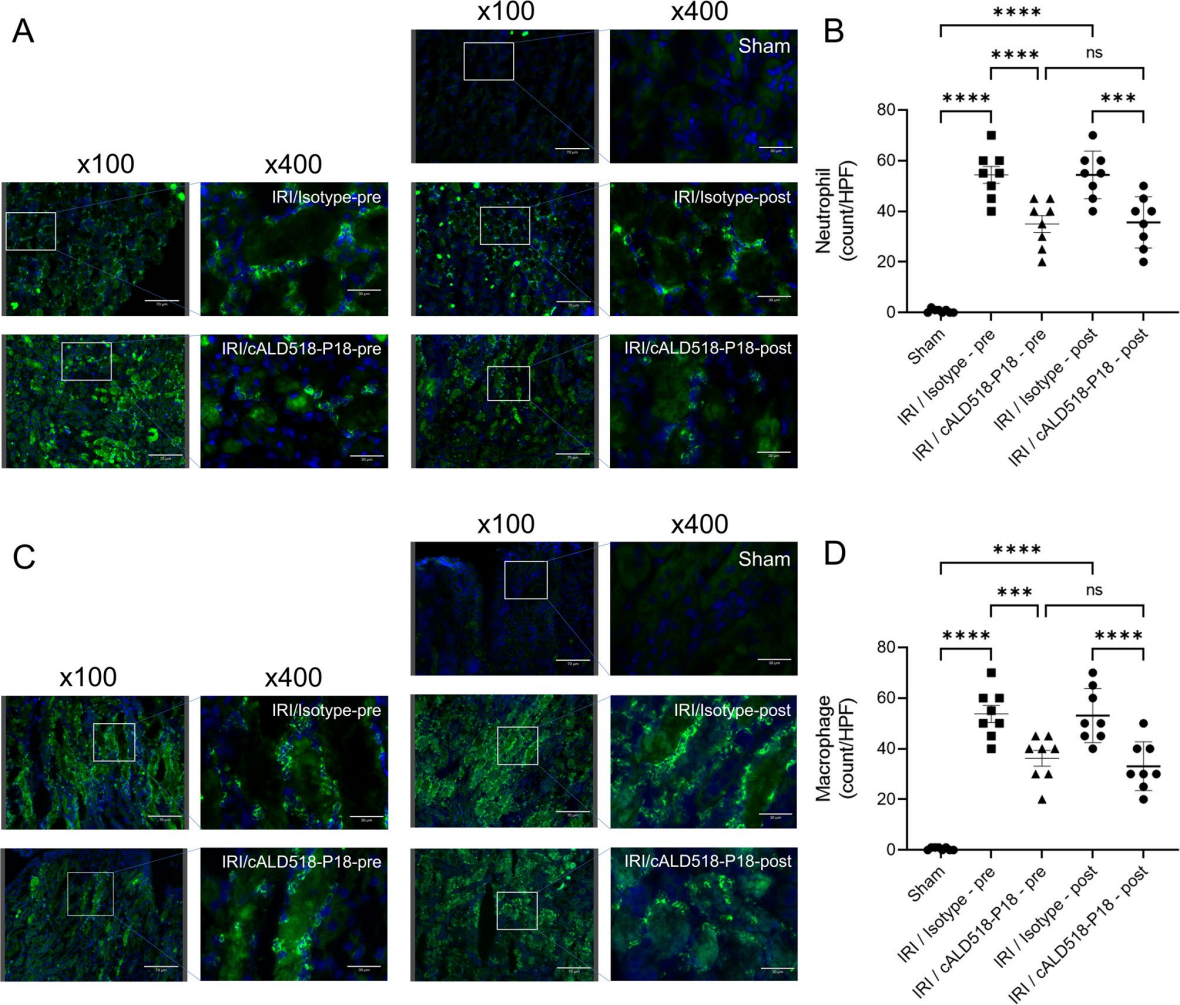
cALD518-P18 treatment reduces innate immune cell infiltration after renal ischemia–reperfusion. Kidney sections were examined by fluorescence microscopy for (**A**, **B**) Ly-6G + neutrophils and (**C**, **D**) F4/80 + macrophages at 24 h post-reperfusion. (**A**, **C**) Representative images for each experimental group at low (× 100, scale bars: 75 µm) and high magnifications (× 400, scale bars: 30 µm). Square boxes in low-magnification images indicate the areas captured at high magnification. (**B**, **D**) Quantification of infiltration expressed as mean cells per high-power field (HPF). IRI/cALD518-P18 treatment attenuated recruitment of both (**B**) neutrophils and (**D**) macrophages, regardless of administration timing. Data are presented as mean ± SEM (n = 8 per group). Statistical significance was determined by one-way ANOVA followed by uncorrected Fisher’s LSD test to perform pre-dedined, planned comparisons (***p < 0.001, ****p < 0.0001; ns, not significant).

Renal IR triggers an acute inflammatory response involving the recruitment of innate immune cells that contribute to tissue damage. Quantification of neutrophils (Fig. 3A,B) and macrophages (Fig. 3C,D) by fluorescence microscopy revealed minimal presence of these cells in the left kidneys of sham-operated mice. A significant infiltration of neutrophils was observed in the left renal cortex of mice in the IRI/Isotype pre- and post- treatment groups (Fig. 3B, both *p* < 0.0001 versus Sham). A similar pattern was observed for macrophage infiltration (Fig. 3D, both *p* < 0.0001 versus Sham). Treatment with cALD518-P18 either before or after ischemia significantly reduced the number of infiltrating neutrophils (Fig. 3B, *p*  < 0.0001 and *p* < 0.001 versus respective IRI/Isotype controls) and macrophages (Fig. 3D, *p*  < 0.001 and *p* < 0.0001 versus respective IRI/Isotype controls).

### cALD518-P18 attenuates endothelial cell activation, glycocalyx shedding and complement activation associated with renal IRI

**Figure 4:**
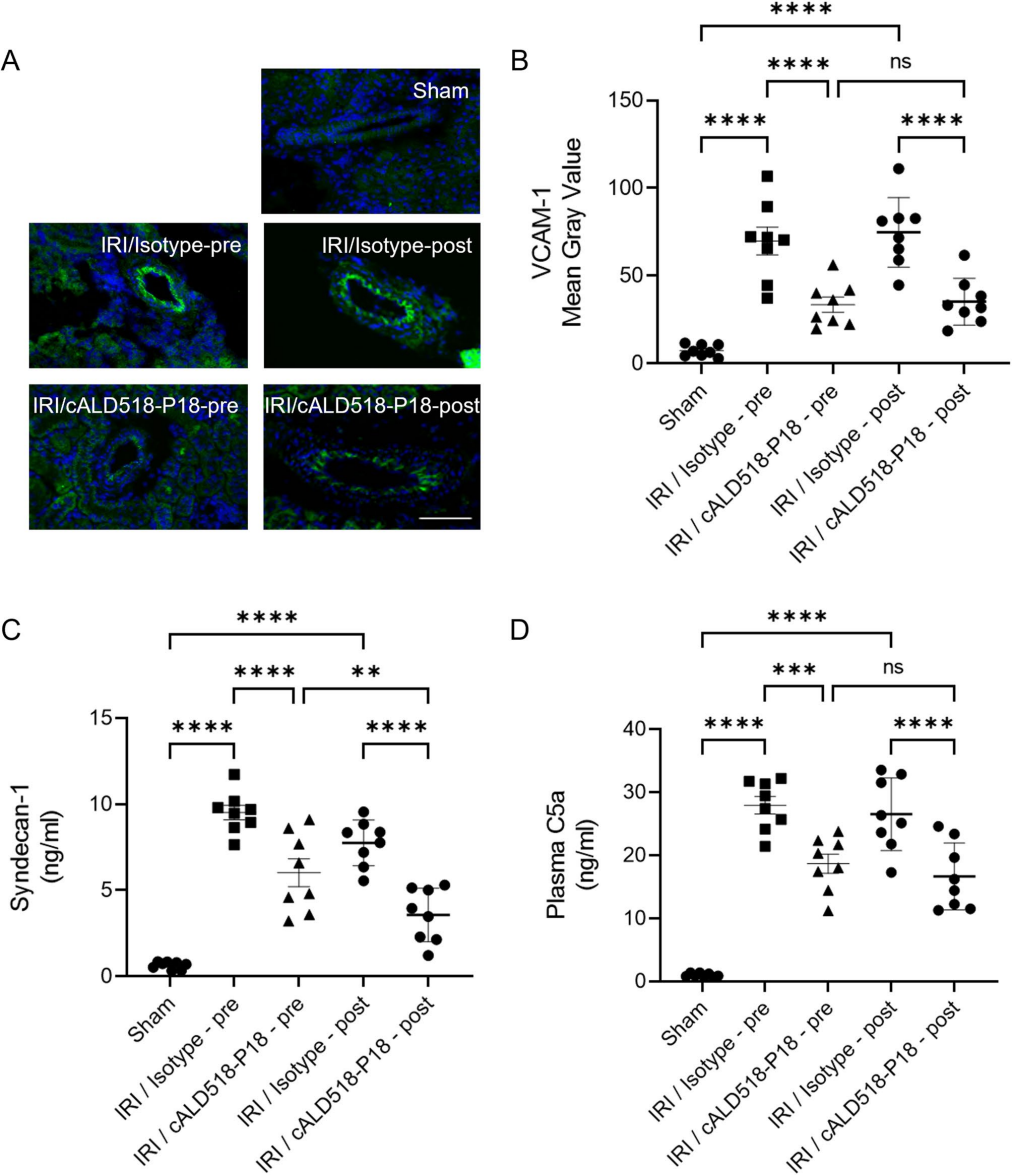
cALD518-P18 treatment reduces endothelial activation, glycocalyx shedding and complement activation associated with renal IRI. (**A**, **B**) Kidney sections were examined for expression of the endothelial activation marker VCAM-1 by immunofluorescence staining and ImageJ analysis. (**A**) Representative images of VCAM-1 staining (scale bar: 30 μm) and (**B**) quantification of mean fluorescence intensity. (**C**, **D**) Plasma levels of (**C**) syndecan-1, (a marker of glycocalyx shedding) and (**D**) the activated complement component C5a measured by ELISA. cALD518-P18 treatment before or after ischemia significantly reduced VCAM-1 expression, plasma syndecan-1 and C5a at 24 h. Data are presented as mean ± SEM (n = 8 per group). Statistical significance was determined by one-way ANOVA followed by uncorrected Fisher’s LSD test to perform pre-dedined, planned comparisons (**p < 0.01, ***p < 0.001, ****p < 0.0001; ns, not significant).

Activation and injury of vascular endothelial cells plays an important role in the pathogenesis of renal IRI^22^. Kidneys from IRI/Isotype mice treated either before or after ischemia displayed strong fluorescence staining for VCAM-1 (Fig. 4A,B), indicating endothelial cell activation. Quantification of staining confirmed that this increase was significant (Fig. 4B, IRI/Isotype-pre and -post both *p* < 0.0001 versus Sham). Kidneys from IRI/cALD518-P18 mice treated either before or after ischemia showed significantly reduced VCAM-1 staining (Fig. 4B, both *p* < 0.0001 versus respective IRI/Isotype controls), indicating that neutralizing IL-6 with cALD518-P18 attenuated endothelial cell activation in this model.

The endothelial glycocalyx is crucial for maintaining vascular integrity, and its loss can lead to increased vascular permeability and further renal damage^23^. We therefore examined plasma levels of the glycocalyx component syndecan-1 as a marker of endothelial cell injury. In addition, we measured complement C5a in plasma as a marker of activation of the complement terminal pathway. IRI/Isotype mice treated either before or after ischemia showed substantial increases in syndecan-1 (Fig. 4C, both *p* < 0.0001 versus Sham) and C5a (Fig. 4D, both *p* < 0.0001 versus Sham), indicating significant shedding of the endothelial glycocalyx and activation of complement in response to IR. IRI/cALD518-P18 mice treated before or after ischemia showed significantly lower levels of syndecan-1 (Fig. 4C, both *p* < 0.0001 versus respective IRI/Isotype controls) and C5a (Fig. 4D, *p*  < 0.001 and *p* < 0.0001 versus respective IRI/Isotype controls). These results indicate that IL-6 blockade with cALD518-P18 reduces endothelial injury and complement activation associated with renal IRI.

### cALD518-P18 reduces pSTAT3 signalling and gene expression of the tubular stress marker NGAL

**Figure 5:**
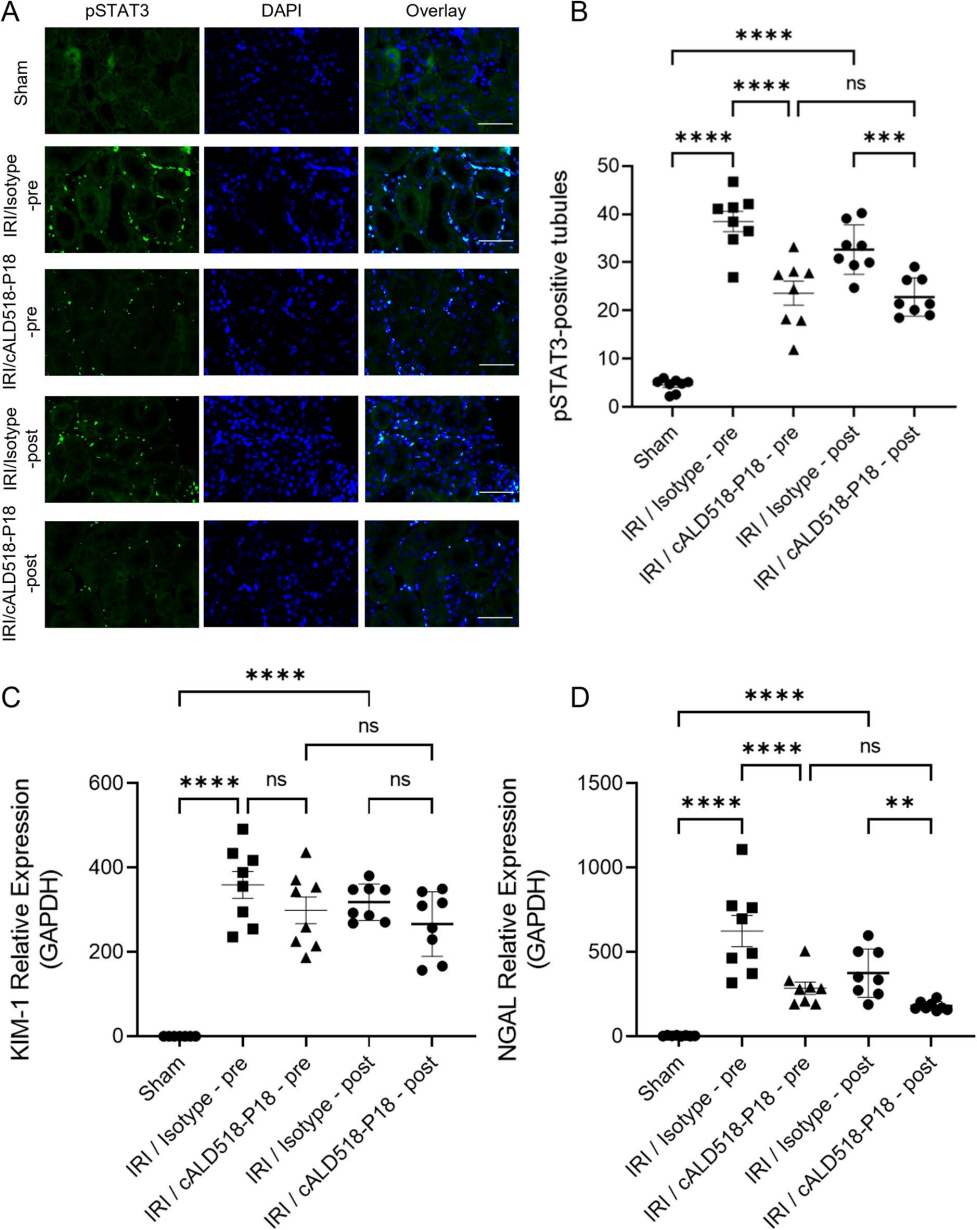
cALD518-P18 treatment dampens activation of the STAT3 pathway and reduces gene expression of the tubular stress marker NGAL after renal ischemia–reperfusion. (**A**, **B**) Kidney sections were examined for expression of phosphorylated STAT3 (pSTAT3) by immunofluorescence staining. (**A**) Representative fluorescence images of pSTAT3 localization in kidney sections (scale bars: 30 μm). (**B**) Quantification of the number of nuclear pSTAT3-positive tubules per field at 24 h post-reperfusion; cALD518-P18 treatment either before or after ischemia significantly reduced STAT3 activation. (**C**, **D**) Gene expression of the injury markers (**C**) KIM-1 and (**D**) NGAL was quantified by RT-PCR. While KIM-1 levels remained elevated, reflecting initial hypoxic structural damage, NGAL expression, a marker of active tubular stress, was significantly attenuated in both cALD518-P18 treatment groups. Data are presented as mean ± SEM (n = 8 per group). Statistical significance was determined by one-way ANOVA followed by uncorrected Fisher’s LSD test to perform pre-dedined, planned comparisons (*p < 0.05, **p < 0.01, ***p < 0.001, ****p < 0.0001; ns, not significant).

Activation of the STAT3 signalling pathway is associated with acute pro-inflammatory responses^24^. To assess JAK/STAT3 pathway activation, pSTAT3 was measured at 24 h post-perfusion using fluorescence staining and image analysis (Fig. 5A,B). The resulting images showed a distinct pattern of pSTAT3 expression, primarily localized to the nuclei of tubular epithelial cells, characterized by discrete, focal FITC-positive immunofluorescence that exhibited precise spatial co-localization with the DAPI nuclear counterstain (Fig. 5A). Kidneys from IRI/Isotype mice treated either before or after ischemia showed a significant increase in pSTAT3-positive tubules (Fig. 5B, both *p* < 0.0001 versus Sham), indicating activation of STAT3 by IR. IRI/cALD518-P18 mice treated either before or after ischemia exhibited significantly reduced pSTAT3-positve tubules (Fig. 5B, p < 0.0001 and *p* < 0.001 versus respective IRI/Isotype controls), indicating that IL-6 blockade effectively diminished STAT3 signalling associated with renal IRI.

To further evaluate the impact of IL-6 neutralization on molecular markers of renal injury, we measured the mRNA expression of kidney injury molecule-1 (KIM-1) and neutrophil gelatinase-associated lipocalin (NGAL) in kidney tissue 24 h after reperfusion. Relative to Sham-operated controls, IRI/Isotype mice exhibited a dramatic induction of both KIM-1 and NGAL mRNA levels (Fig. 5C,D, both *p* < 0.0001). While KIM-1 mRNA expression remained elevated and was not significantly altered by cALD518-P18 treatment (Fig. 5C), reflecting established structural damage from the initial hypoxic insult, NGAL mRNA expression, a marker of active tubular stress, was significantly downregulated in both the pre-ischemia (*p* < 0.01) and post-ischemia (*p* < 0.05) cALD518-P18 treatment groups compared to their respective isotype controls (Fig. 5D). These molecular findings are consistent with the observed improvements in renal function and the significant reduction in histological tubular injury scores and pSTAT3 signalling.

### cALD518-P18 variably affects circulating cytokines while reducing local inflammation in the kidney

**Figure 6:**
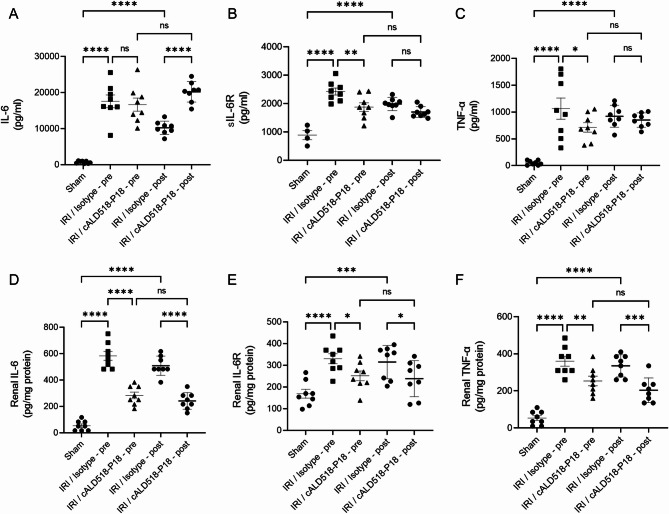
cALD518-P18 treatment has variable effects on levels of circulating and local IL-6, sIL-6R and TNF-α after renal ischemia–reperfusion. Plasma samples collected 24 h after reperfusion were analyzed by ELISA for IL-6, sIL-6R, and TNF-α (**A**–**C**). cALD518-P18 treatment before ischemia reduced sIL-6R (**B**) and TNF-α (**C**), but had no effect on IL-6 (**A**). cALD518-P18 treatment after ischemia increased IL-6 (**A**), but had no effect on sIL-6R (**B**) or TNF-α (**C**). Renal tissue lysates were analyzed by ELISA for IL-6, IL-6R, and TNF-α (**D**–**F**). cALD518-P18 treatment before or after ischemia significantly reduced IL-6 (**D**), IL-6R (**E**), and TNF-α (**F**) at 24 h post-reperfusion. Data are presented as mean ± SEM (n = 4–8 per group). Statistical significance was determined by one-way ANOVA followed by uncorrected Fisher’s LSD test to perform pre-dedined, planned comparisons (*p < 0.05, **p < 0.01, ****p < 0.0001; ns, not significant).

We next analyzed plasma levels of IL-6, sIL-6R, and the pro-inflammatory cytokine TNF-α at 24 h. Interestingly, some differences were observed depending on the timing of treatment. With pre-ischemia treatment, IRI/Isotype mice showed significant increase in IL-6 (Fig. 6A), sIL-6R (Fig. 6B), and TNF-α (Fig. 6C) (all *p* < 0.0001 versus Sham), indicating a robust inflammatory response including IL-6 trans-signalling. cALD518-P18 given pre-ischemia reduced sIL-6R (Fig. 6B, *p*  < 0.01 versus IRI/Isotype-pre) and TNF-α (Fig. 6C, *p*  < 0.05 versus IRI/Isotype-pre), but did not significantly affect IL-6 (Fig. 6A). With post-ischemia treatment, IL-6, sIL-6R and TNF-α were again all significantly increased in IRI/Isotype mice versus Sham (all *p* < 0.0001). Notably, the magnitude of the increase in IL-6 in the IRI/Isotype-post group was lower than that in the IRI/Isotype-pre group (Fig. 6A). cALD518-P18 post-ischemia had no effect on sIL-6R (Fig. 6B) or TNF-α (Fig. 6C) levels compared to isotype control, but significantly increased IL-6 (Fig. 6A, *p*  < 0.0001 versus IRI/Isotype-post). This increase appeared to be related to the lower level of IL-6 in the IRI/Isotype-post group (mean approx. 10,000 pg/ml versus approx. 17,500 pg/ml for the IRI/Isotype-pre group (Fig. 6A).

To determine if the effects on circulating cytokines reflected the local inflammatory environment within the injured organ, we measured IL-6, IL-6R/sIL-6R, and TNF-α levels in kidney tissue lysates. Compared to Sham, kidneys from IRI/Isotype mice treated either before or after ischemia showed significant increases in IL-6 (Fig. 6D, both *p* < 0.0001), IL-6R/sIL-6R (Fig. 6E, *p*  < 0.001 and *p* < 0.0001, respectively), and TNF-α (Fig. 6F, both *p* < 0.0001), indicating a strong inflammatory response involving IL-6 trans-signalling. In contrast to the complex findings in plasma, treatment with cALD518-P18 resulted in a significant reduction of all three markers within the kidney tissue. IRI/cALD518-P18 mice treated either before or after ischemia revealed significantly lower levels of IL-6 (Fig. 6D, both *p* < 0.0001 versus respective IRI/Isotype controls), IL-6R/sIL-6R (Fig. 6E, both *p* < 0.05 versus respective IRI/Isotype controls), and TNF-α (Fig. 6F, *p*  < 0.01 and *p* < 0.001 versus respective IRI/Isotype controls). This demonstrates that while systemic cytokine measurements can be complex, cALD518-P18 suppresses the local inflammatory milieu within the target organ.

## Discussion

The absence of effective clinical therapies targeting renal IRI presents a significant challenge in kidney transplantation, particularly with the increased deployment of donation after circulatory death (DCD) and associated longer warm ischemia times. IL-6 is a key player in the inflammatory process of IRI^25^. Elevated IL-6 has been correlated with the severity of renal IRI, with high pre-transplant or early post-transplant levels serving as potential biomarkers for predicting delayed kidney graft function^26^. Targeting IL-6 in the peri-transplant period is therefore a logical protective approach. However, pharmacological modulation of IL-6 signalling has produced mixed outcomes in a range of mouse models of warm renal IRI^11–14^. Our goal in this study was to use the best tools available to us—a reliable, proven model^15–18^, and a high affinity anti-IL-6 mAb—to address the question of whether neutralization of IL-6 is effective in attenuating renal IRI. Our results demonstrated that cALD518-P18, given either shortly before ischemia or immediately after reperfusion, dampened inflammation and significantly reduced renal IRI markers, including serum creatinine, urea, and the acute tubular stress marker NGAL.

A crucial component of our study is the specific therapeutic agent used. The antibody cALD518-P18 is a chimeric variant of ALD518-P18, which is itself derived from clazakizumab and has been specifically engineered with CDR mutations for enhanced binding and neutralization of murine IL-6. It combines human variable domains with mouse IgG1 constant regions, matching the isotype control. Critically, it incorporates an N297A mutation in the Fc region to eliminate its effector functions. This is a key feature as it ensures that the observed therapeutic benefits are due to specific IL-6 neutralization, not non-specific Fc-mediated anti-inflammatory effects, thereby validating our comparison to the effector-competent MOPC-21 isotype control. The 40 mg/kg dose used was established through a dose–response study (Fig. 1A), which identified the optimal therapeutic window for functional recovery in this model.

We addressed the mechanism of injury in this model by measuring the level of circulating sIL-6R, which mediates pro-inflammatory IL-6 trans-signalling^3^, and kidney pSTAT3, which is a downstream marker of trans-signalling. Indeed, both sIL-6R and tubular pSTAT3 staining were significantly increased in control mAb-treated mice at 24 h post-IR, as were IL-6 itself and another pro-inflammatory cytokine, TNF-α. While these findings are consistent with the activation of the IL-6 trans-signalling pathway, we acknowledge that our study does not definitively distinguish between free and antibody-bound IL-6, nor does it directly measure gp130 activation. However, pSTAT3 immunofluorescence protocol revealed nuclear localization of the signal, which was significantly abrogated by cALD518-P18 treatment. This provides direct evidence of target engagement and effective signalling blockade at the tissue level, confirmed by the precise co-localisation with DAPI conterstaining. Abrogation of tubular pSTAT3 by cALD518-P18 aligns with genetic studies confirming IL-6 as a central driver of renal IRI^11,12^. This pharmacological blockade effectively targets the STAT3α isoform, recently identified as the pivotal mediator of AKI progression^27^, providing a dominant protective effect by suppressing the acute pro-inflammatory surge. To further evaluate the localized impact, we examined VCAM-1, a critical marker of endothelial activation and injury, and NGAL, a sensitive 'real-time’ biomarker of active tubular epithelial cell stress. Although these downstream changes (pSTAT3, NGAL, VCAM-1) are correlative, the use of a high-affinity neutralizing antibody provides a strong indication of the pathogenic role of the IL-6 axis in this model.

Comparing the systemic versus local cytokine data provides mechanistic insight. In the systemic circulation, cALD518-P18 treatment significantly reduced sIL-6R and TNF-α when given pre-ischemia, but did not reduce total plasma IL-6. We suggest two possible explanations: a rebound effect, as previously observed with anti-IL-6R therapies such as tocilizumab^28,29^, although this is unlikely within 24 h; the more plausible explanation is a pharmacokinetic artifact, failure of the IL-6 ELISA to distinguish between free and cALD518-P18-bound IL-6. The antibody-cytokine complex has a much longer circulatory half-life, leading to an apparent accumulation of total IL-6 in plasma, despite the free, biologically active form being effectively neutralized. In the absence of serial IL-6 measurements, we cannot distinguish between these possibilities. Another intriguing finding that we cannot yet explain was the effect of timing of treatment on the degree of induction of IL-6 in the control mAb-treated groups; post-ischemia treatment resulted in lower level of IL-6 than pre-ischemia treatment (Fig. 6A).

In contrast to the plasma results, cALD518-P18 treatment reduced the local tissue concentrations of IL-6, sIL-6R, and TNF-α (Fig. 6D–F). While we have not provided direct evidence of antibody localization within the renal tissue, the localized reduction of these markers suggests a proposed compartmentalised renal effect. We propose that cALD518-P18 sequesters IL-6 produced by the kidney in response to IR, preventing it from engaging its receptor and initiating trans-signaling. This local blockade blunts the cytokine’s pathogenic actions on key resident cell types, preventing neutrophil recruitment, reducing endothelial cell activation, preserving the endothelial glycocalyx, inhibiting complement activation, and blocking activation of the JAK/STAT3 pathway in tubular cells. Future studies tracking fluorescently labeled antibodies could confirm if the therapeutic reaches the renal interstitial space effectively, which would support strategies like ex vivo normothermic perfusion of donor kidneys.

The observation of a differential response between KIM-1 and NGAL expression further supports this model of localized protection against acute inflammatory stress. KIM-1 tracks established structural damage that originates during the primary ischemic (hypoxic) phase^30,31^. While IL-6 blockade successfully mitigates the active inflammatory injury (reflected in improved injury scores and NGAL), KIM-1 levels likely reflect the initial hypoxic insult and the resulting luminal debris, which take longer to resolve despite reductions in creatinine and urea^31,32^. As NGAL is a sensitive "real-time" indicator of acute tubular cell stress^33,34^, its reduction aligns with the improvement in histological injury scores and the dampening of pSTAT3 signalling, suggesting that anti-IL-6 therapy effectively mitigates the secondary, reperfusion-induced injury cascade even when initial hypoxic damage has occurred.

Some differences were observed when cALD518-P18 was given before or after ischemia. Notably, post-ischemia i.v. treatment appeared to be more protective of kidney function than pre-ischemia i.p. treatment (Fig. 1B,C). This suggests that while both treatment regimens reduce cellular infiltration and activation, the immediate bioavailability of i.v. administered drug at the time of reperfusion may provide a pharmacokinetic advantage that translates into superior organ protection, and has potential clinical implications beyond transplantation. Kidney IRI during transplantation is an event that can be anticipated and is therefore a feasible target for pre-transplant protective therapies. However, there are other clinical scenarios in which ischemic AKI is not predictable and is therefore difficult to treat pre-emptively. One example is AKI in ICU patients, a highly prevalent complication which is frequently caused by and/or worsened by hypoperfusion of the kidney^35^. Targeting IL-6 may offer a potential treatment strategy after ischemia in this context.

This study has several limitations that must be acknowledged. Firstly, our analysis was restricted to a single 24-h endpoint, which captures the peak of acute injury but provides no information on the longer-term consequences; we have previously observed that extending the endpoint beyond 24 h results in an unacceptable mortality rate in control-treated mice (data ot shown). However, since the severity of the initial IRI is a key determinant of progression to chronic kidney disease (CKD)^36^, it is plausible that cALD518-P18 treatment could reduce subsequent fibrosis. While KIM-1 remained elevated at 24 h, likely reflecting initial hypoxic structural damage, the significant downregulation of NGAL suggests a reduction in active cellular stress. Conversely, it has been suggested that STAT3 activation promotes repair of kidney injury by upregulating anti-apoptotic genes and growth factors^27^, but cALD518-P18 reduced pSTAT3 levels in the kidney, potentially compromising tissue repair. Secondly, our study was performed in a non-transplant IRI model, to specifically isolate the effects of warm ischemia. This model is highly relevant for DCD transplantation but lacks the influence of cold ischemia and the alloimmune response, which would create a more complex inflammatory environment. Third, the use of an effector-defective therapeutic antibody with an effector-competent isotype control antibody (MOPC-21) is a limitation, but the strong protective data across multiple measures suggests that IL-6 neutralization is the primary mechanism of action.

In conclusion, we provide evidence for the efficacy of cALD518-P18 treatment in reducing acute renal injury and dysfunction associated with IRI. While the mechanistic conclusions regarding compartmentalization and trans-signalling remain hypotheses to be further validated, the consistent improvement across functional and histological markers is compelling. Future research utilizing CRISPR-Cas9 gene editing or cell-specific inducible knockouts will be essential to definitively establish causality and dissect the complex, tissue-specific nuances of the IL-6–STAT3 axis^37–39^. Our future work will use our established mouse model of IRI-induced CKD to investigate the long-term effects of this acute IL-6 blockade on renal fibrosis at 28 days^40^. Furthermore, these promising results provide a strong rationale for advancing these studies into more complex, clinically relevant models of kidney transplantation to determine if this targeted therapy can improve graft survival in the face of both IRI and alloimmunity.

## Materials and methods

### Animals

10- to 12-week-old male C57BL/6 WT mice were purchased from the Animal Resources Centre in Canning Vale, Western Australia, and were housed in the Bioresources Centre at St. Vincent’s Hospital Melbourne. All procedures were approved by the Animal Ethics Committee (AEC Reference number: 024/20) of St. Vincent’s Hospital Melbourne and adhered to relevant ethical guidelines and regulations. This study complies with the ARRIVE guidelines (Animal Research: Reporting in Vivo Experiments).

### Warm renal IRI

Unilateral warm renal IRI was induced in mice aged 10 to 12 weeks, employing established protocols^16^. The mice were anesthetized by intraperitoneal injection of ketamine (100 mg/kg) and xylazine (15 mg/kg), with body temperature maintained at 35.5–36.5 °C using a heating pad. Prior to surgery, analgesia was provided via a subcutaneous injection of buprenorphine (0.1 mg/kg). A midline abdominal incision exposed the kidneys, allowing for blunt dissection of the renal pedicles. After right nephrectomy, ischemia was induced by occluding the left renal pedicle with a microvascular clamp (Roboz, Rockville, MD) for 22 min, after which reperfusion was confirmed by visual inspection. The surgical wound was closed, and each mouse received an infusion of 200 µl warm saline into the abdominal cavity. Sham-operated mice underwent the same procedure, except without left renal pedicle clamping. Recovery involved placement on a heat pad and administration of atipamezole (0.25 mg/kg) to expedite recovery from anesthesia. 24 h post-reperfusion, mice were re-anesthetized with ketamine/xylazine, exsanguinated via the inferior vena cava, and blood and kidney samples were collected for subsequent analysis.

### Antibody treatment

cALD518-P18 and an irrelevant isotype-matched control mAb (MOPC-21, mouse IgG1κ) were supplied by CSL Ltd. (Parkville, VIC, Australia). An initial dose–response study was performed to establish a therapeutic dose as described in the Results section. For the experimental groups, 40 mg/kg (in 200–220 µL volume) of either cALD518-P18 or the isotype control antibody was administered by one of two routes: intraperitoneally (i.p.) 30 min before ischemia, or i.v. immediately after ischemia.

### Experimental groups

There were 5 experimental groups (n = 8 mice per group): (1) pre-ischemia treatment with cALD518-P18 (IRI/cALD518-P18-pre); (2) pre-ischemia treatment with isotype control antibody (IRI/Isotype-pre); (3) post-ischemia treatment with cALD518-P18 (IRI/cALD518-P18-post); (4) post-ischemia treatment with isotype control antibody (IRI/Isotype-post); and (5) sham control (Sham).

### Assessment of renal function

Renal function was evaluated by measuring serum creatinine using a kinetic colorimetric assay based on the Jaffé method, performed on a COBAS Integra 400 Plus analyzer (Roche, Castle Hill, Australia), according to the manufacturer’s guidelines. Serum urea levels were determined using the Urea Assay Kit STA-382 (Cell Biolabs, San Diego, CA) following the provided instructions.

### Histopathology

Kidney biopsies were fixed in 10% formaldehyde and embedded in paraffin. Sections were cut at 4 μm and stained with hematoxylin–eosin (H&E) according to standard protocols. H&E-stained sections were then analyzed to assess renal tubular injury by calculating the percentage of injured or necrotic tubules relative to the total number of tubules in each section. A blinded, trained veterinary pathologist (F.M.) assessed twelve randomly selected corticomedullary fields per sample (four from each pole: upper, mid, and lower; 400 × magnification) for signs of tubular injury, including epithelial cell degeneration, sloughing, dilation, and cast formation. The average tubular injury percentage was calculated for each sample.

### Renal tissue lysate preperation

Snap-frozen tissue was homogenized in ice-cold radioimmunoprecipitation assay (RIPA) buffer supplemented with a protease inhibitor cocktail (1:100 dilution, 5872S, Cell Signaling Technology, Danvers, MA). The homogenates were centrifuged, and the resulting supernatant was collected. Protein concentration was quantified using the Bradford Protein Assay (23,236, Thermo Fisher Scientific, Waltham, MA). Protein concentrations were adjusted to 1 mg/mL and aliquots were stored at –80 °C until further analysis.

### ELISA for syndecan-1, complement C5a, IL-6, sIL-6R, and tumor necrosis factor-α (TNF-α)

Plasma samples were analyzed using commercial ELISA kits for mouse syndecan-1 (75-138MS-S10, Alpco, Salem, NH), and C5a (DY2150, R&D Systems, Minneapolis, MN) following the manufacturer’s instructions. Both, plasma and renal lysates were analyzed by ELISA for IL-6 (900-K50; Thermo Fisher Scientific), IL-6R/sIL-6R (EMIL6RA, Thermo Fisher Scientific), and TNF-α (DY410; R&D Systems) according to the manufacturer’s instructions. Absorbance was read at 450/540 nm using a FLUOstar Omega microplate reader (BMG Labtech, Offenburg, Germany).

### Immunofluorescence

Fresh-frozen tissue sections (5 μm) were fixed with acetone and incubated with various antibodies, including rat anti-mouse vascular cell adhesion molecule (VCAM)-1 Alexa 488 (1:50 dilution, clone MVCAM A, MCA2297, Bio-Rad), rat anti-mouse Ly-6G FITC (1:50, 1A8, 127,605, BioLegend, San Diego, CA), rat anti-mouse F4/80 FITC (1:50, BM8, 123,107, BioLegend) or rabbit anti-pSTAT3 (Tyr705, 1:200, D3A7, 9145, Cell Signaling Technology, Danvers, MA), overnight at 4 °C. For pSTAT3 staining, formalin-fixed paraffin-embedded tissue Sects. (4 μm) were used. Following antigen retrieval, sections were incubated with rabbit anti-pSTAT3 Tyr705 (1:50, 9145, Cell Signaling Technology, Danvers, MA) for 2 h at room temperature. To ensure signal specificity, negative controls (omission of primary antibodies) and isotype-matched control (1:50–1:200, rabbit mAb IgG, 3900, Cell Signalling Technology) were processed in parallel with the experimental samples. Following 3 washes, pSTAT3 antibody was detected using goat anti-rabbit IgG Alexa Fluor 488 (1:500, A11070, Thermo Fisher Scientific) for 60 min at room temperature.

Slides were analyzed using an ECHO Revolve R4 hybrid microscope (ECHO, San Diego, CA), and quantification of staining was performed using ImageJ software version 10.2 by observers blinded to the treatment groups. Measurement metrics included mean gray values for VCAM-1 fluorescence intensity in blood vessels, and cell counts per high-power field (HPF) for Ly-6G and F4/80, which are markers of neutrophils and macrophages, respectively. For quantification of pSTAT3, the number of tubules showing positive pSTAT3 staining was counted in ten random corticomedullary field per sample, and expressed as the average number of pSTAT3-positive tubules per field.

### Quantitative reverse transcriptase PCR

Kidney segments (one-eighth) were preserved in RNAlater at 4 °C for 24–48 h before long-term storage at -80 °C. Total RNA extraction was performed with the ReliaPrep RNA Tissue Miniprep System (Promega Australia) as per the manufacturer’s protocol. RNA concentration and purity were assessed using a FLUOstar Omega microplate reader. For cDNA synthesis, 1.0 mg of total RNA was combined with 0.5 mg oligo(dT) and 1 mg random primers in a 50-μl volume and heated to 70 °C for 10 min. This was followed by the addition of a master mix containing 10 mM dNTPs, 300 U SuperScript III, 60 U RNaseOUT, 0.1 M DTT, and 5 × first-strand buffer. The reverse transcription reaction was carried out at 42 °C for 60 min and terminated at 70 °C for 10 min, with the resulting cDNA kept at -20 °C. Gene expression was quantified via real-time PCR utilizing TaqMan Universal PCR Master Mix and specific TaqMan assays for KIM-1 (Havcr1: Mm00506686_m1), NGAL (Lcn2: Mm01324470_m1), and GAPDH (Gapdh: Mm99999915_g1) on a 7500Fast Real-Time PCR System.

### Statistical analysis

Statistical evaluation and data visualization were performed using Prism version 9.5.1 (GraphPad, San Diego, CA). Results are represented as mean ± standard error of the mean (SEM). Groups were compared by one-way ANOVA with uncorrected Fisher’s Least Significance Difference (LSD) test. To test our primary hypotheses, we performed pre-defined planned comparisons rather than an exploratory all-pairs analysis. In a single LSD analysis, the following five biologically relevant comparisons were performed: (i) Sham vs. IRI/Isotype-pre and (ii) IRI/Isotype-pre vs. IRI/cALD518-P18-pre to establish injury and pre-ischemia efficacy; (iii) Sham vs. IRI/Isotype-post and (iv) IRI/Isotype-post vs. IRI/cALD518-P18-post to establish injury and post-ischemia efficacy; and (v) IRI/cALD518-P18-pre vs. IRI/cALD518-P18-post to compare the two therapeutic regimens. Because these comparisons were specified a priori and were not part of an all-pairs exploratory analysis, *p*-values were not corrected for multiple comparisons, in accordance with standard statistical practice for planned analyses^41,42^. A significance threshold of *p* < 0.05 was set to determine statistical significance.

## Data Availability

The data used and analyzed during this current study are available from the corresponding author, Anjan K Bongoni, upon reasonable request via e-mail at anjan.bongoni@svha.org.au.

## Abbreviations

AKI: Acute kidney injury
CKD: Chronic kidney disease
CDR: Complementarity-determining regions
IL-6: Interleukin-6
i.p.: Intraperitoneally
i.v.: Intravenously
IRI: Ischemia-reperfusion injury
KIM-1: Kidney injury molecule-1
mAb: Monoclonal antibody
NGAL: Neutrophil gelatinase-associated lipocalin
pSTAT3: Phosphorylated signal transducer and activator of transcription 3
sIL-6R: Soluble IL-6 receptor
TNF: Tumor necropsis factor
VCAM-1: Vascular cell adhesion molecule-1
WT: Wild-type
